# Medical Scribes in an Orthopedic Sports Medicine Clinic Improve Productivity and Physician Well-Being

**DOI:** 10.1016/j.asmr.2022.02.003

**Published:** 2022-04-08

**Authors:** Jordan R. Pollock, M. Lane Moore, Aaron C. Llanes, Joseph C. Brinkman, Justin L. Makovicka, Donald L. Dulle, Nathaniel B. Hinckley, Anthony Barcia, Matthew Anastasi, Anikar Chhabra

**Affiliations:** a Mayo Clinic Alix School of Medicine, Scottsdale Arizona, U.S.A.; b University of Arizona School of Medicine, Phoenix, Arizona, U.S.A.; c Department of Orthopedic Surgery, Mayo Clinic, Rochester, Minnesota, U.S.A.; d Department of Family Medicine, Sports Medicine, Mayo Clinic, Rochester, Minnesota, U.S.A.

## Abstract

**Purpose:**

The purpose of this study is to examine the effects of scribe use on physicians, nurses, and patients in an orthopaedic sports medicine clinic.

**Methods:**

Surveys containing validated outcome measures relating to physician well-being and workplace satisfaction, among other variables, were used to assess the influence of medical scribes on clinic function. These surveys were collected for 8 months from all patients, nurses, and orthopaedic surgeons working in one orthopaedic sports medicine clinic. Time during a half-day clinic (i.e., 20 or more patients) was documented by surgeons after the last patient was seen.

**Results:**

The average time spent per half day of clinic was 104 minutes on nonscribe days and 25 minutes on scribe days. Additionally, the time spent documenting encounters per half day of clinic was 87 minutes on average without scribes and 26 minutes on average with scribes. The average surgeon single assessment numeric evaluation (SANE) score was 48.1 without scribes, and 89.3 with scribes. The overall assessment of the clinic by nurses was 73.4 out of 100 on average without scribes and 87.7 out of 100 on average with scribes. Patients did not report a significant change in rating of overall experience (4.7/5.0 with scribes and 4.8/5.0 without scribes, (*P* = .27) or wait time between scheduled appointment time and surgeon arrival (15.1 minutes with scribes and 18.1 minutes without scribes; *P* = .12).

**Conclusions:**

We found the use of scribes in a high-volume orthopaedic sports medicine clinic to have a favorable impact on physicians, nurses, and trainees. The use of a scribe also significantly reduced the time required by surgeons for documentation during clinic and at the end of each clinic day. Patients also reported no significant difference in patient clinic experience scores.

**Clinical Relevance:**

Orthopaedic surgeons spend a substantial amount of time on paperwork. The results of this study could provide information on whether the use of a scribe helps to reduce administrative burden on orthopedic surgeons.

## Introduction

Orthopaedic surgery is a time-consuming career. According to a recent survey by the American Association of Medical Colleges (AAMC), orthopaedic surgeons work an average of 57.0 hours per week.^1^ Another study of 152 “highly successful orthopaedic surgeons”, defined as surgeons who are departmental chairs, presidents of major orthopaedic organizations, or editors of major orthopaedic journals, reported an average of 70.3 hours worked per week.^2^ In addition to working long hours, a 2020 survey reported that 37% of orthopaedic surgeons are either burned out, depressed, or both.^3^ The two most highly cited causes of burnout in these surgeons were “too many bureaucratic tasks (e.g., charting, paperwork)”, and “increasing computerization of practice (EHRs),” with 65% and 44% of orthopaedic surgeons reporting these factors, respectively.^3^ The implementation of the Electronic Health Record (EHR) systems has increased the burden of time spent on patient charting, with studies reporting an increase in physician documentation time between 11% and 22%.^4^ This is confounded when considering orthopaedic surgeons spend an average of 13.7 hours per week on paperwork and administration duties beyond their clinical responsibilities.^5^

As physician burnout becomes an increasingly relevant topic, practice adaptations to reduce burnout and increase physician satisfaction and health have become paramount. Several studies have examined the role of scribes and found improvements in physician quality of life, physician burnout, and patient satisfaction.^6^, ^7^, ^8^, ^9^ Additionally, scribes have been shown to reduce the number of hours spent charting by surgeons.^10^ Not only do the physicians themselves benefit from scribes, but the practices and hospitals can also benefit. For example, numerous studies performed in subspecialty clinics, emergency clinics, and primary care settings have shown that the use of medical scribes can increase physician productivity, as well as revenue, while improving physician and patient satisfaction scores.^11^, ^12^, ^13^, ^14^, ^15^, ^16^, ^17^, ^18^, ^19^, ^20^

Physicians, and particularly surgeons, thrive when performing patient care. However, the burden of charting and documenting within an EHR can inhibit high-volume surgeons from maximizing their time with patient**s**. Decreasing the amount of time surgeons spend charting could allow surgeons to fully use their expertise, listening, examining, diagnosing, educating, and treating patients in clinic. With the help of trained experts in documentation, there is a potential to increase patient and surgeon satisfaction, reduce patient wait times, and increase clinic productivity. This is especially important in the digital era, where patient satisfaction and dissatisfaction are publicly available on websites such as Healthgrades, Vitals, and Yelp.^21^

Innovative approaches to shift the physician’s focus back to patient care and away from repetitive documentation are needed. One such approach is the use of medical scribes. Medical scribes are trained personnel who provide physicians with documentation assistance and perform other EHR tasks.^22^ There is a paucity of literature regarding the use of scribes in orthopaedic clinics, particularly in high-volume practices, classified in this study as 20 patients or more per half-clinic day, or 40 patients per full day. The purpose of this study is to examine the effects of scribe use on physicians, nurses, and patients in an orthopaedic sports medicine clinic. We hypothesize the use of scribes in a high-volume orthopaedic sports clinic will decrease documentation time for physicians and nurses, with no associated decrease in patient satisfaction or clinic experience.

## Methods

Our study was deemed exempt by the Institutional Review Board of our institution. Beginning on January 1, 2020, data collection began for three orthopaedic sports medicine physicians, their nurses, and patients via surveys on clinic days with scribes and clinic days without scribes. These participants were surveyed every clinic day. These surveys contained a validated outcome measure to assess physician time utilization and well-being, as well as nursing and patient surveys to assess the influence of medical scribes on clinic function^17^^,^^23^, ^24^, ^25^ (Appendix).

We collected these surveys from orthopaedic surgeons, patients, and nurses during an 8-month time period. Of these 8 months, 6 months were collected from study participants without scribes, and 2 months were collected from participants with scribes. The study group of physicians and nurses was the same throughout the length of the study. Intentionally, the only difference in clinic was the use of scribes or no scribes. Surveys that were incomplete or illegible were excluded. Each surgeon was given their own personal scribe during the scribe period. The scribes were consistent, outsourced scribes trained in orthopaedic surgery, and were implemented in the outpatient clinic. Several local companies provide this service. Notes were completed in real time. The clinic sees a mix of new patients, consults, and follow-ups. The clinic typically sees 20-25 patients per half day of clinic.

### Physician Survey

All orthopaedic surgeons working at the orthopaedic sports medicine clinic were asked on the basis of a 5-point Likert scale with 1 being “strongly disagree” and 5 being “strongly agree” if they felt that they had “adequate time to perform patient education” and if they felt they had “adequate time to teach medical students and trainees.” A Single Assessment Numeric Evaluation (SANE) score was calculated for each surgeon, and surgeons were asked to score their physical well-being, emotional well-being, spiritual well-being, intellectual well-being, and overall well-being on a scale of 1 being worst possible and 10 being best possible.

### Nursing Survey

All nurses working at the sports medicine clinic during the time period of this study were asked a binary answer of “yes” or “no” to “the surgeon was rushed today” as well as “communication outside of the exam room was effective today.” In this sports clinic, each orthopaedic surgeon worked with the same nurse every day. These nurses help room the patient, meet the patient, direct physician clinic flow, help fill orders, help direct the patient out of the clinic, among other tasks. Each nurse was tasked with assigning an overall score as to their assessment of clinic functioning for the day when the surgeon did not have a scribe and when the surgeon had a scribe. The physicians were blinded to nurse responses to maintain confidentiality and to ensure unbiased responses.

### Patient Survey

All patients presenting to the orthopaedic sports medicine clinic during the time period of this study were asked to rate their experience in the clinic on a Likert scale from 1 to 5 with 1 being “worst possible experience” and 5 being “best possible experience.” The time to clinic room was recorded for each patient visit, as was the time between rooming and when the physician first entered the room to begin their encounter. Physicians were blinded to patient responses.

### Statistical Analysis

The collected data were summarized using descriptive statistics such as mean, standard deviation, median, and frequencies as appropriate. A 2-tailed *t*-test was used to assess the differences between the scribe present and scribe absent groups for all continuous variables. Alpha was set to .05. All statistical analysis was completed using Microsoft Excel (Microsoft, Redmond, WA).

## Results

### Surgeon Results

According to 32 physician surveys from 3 surgeons for the 6 months of nonscribe clinic days, the average time spent per half day of clinic documenting after the last patient was seen was 104 minutes on average. On a scale of 1 (strongly disagree) to 5 (strongly agree), surgeons reported a 2.2 out of 5 score for having “adequate time to perform patient education in clinic today,” and 2.0 out of 5 score for “I had adequate time to teach medical students and trainees today.” The time spent documenting encounters throughout each half-day of clinic was estimated to be 87 minutes on average. The average surgeon single assessment numeric evaluation (SANE) score was 48.1. The average score relating to physician health was physical well-being (6.5/10), emotional well-being (6.5/10), spiritual well-being (7.0/10), intellectual wellbeing (7.8/10), and overall well-being (7.1/10).

**Fig 1:**
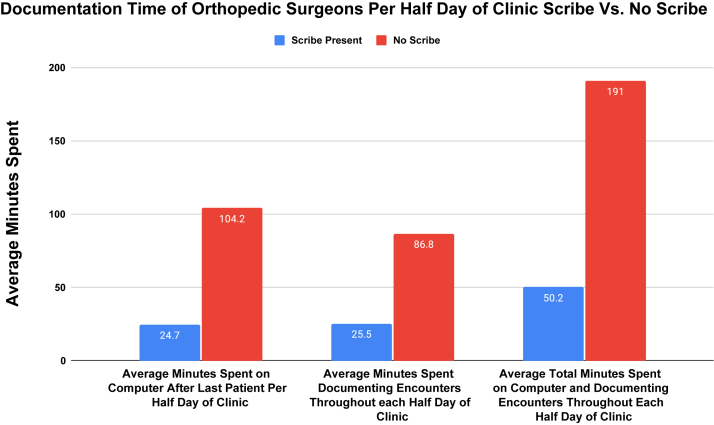
Documentation time of orthopaedic surgeons with scribes and without scribes.

**Fig 2:**
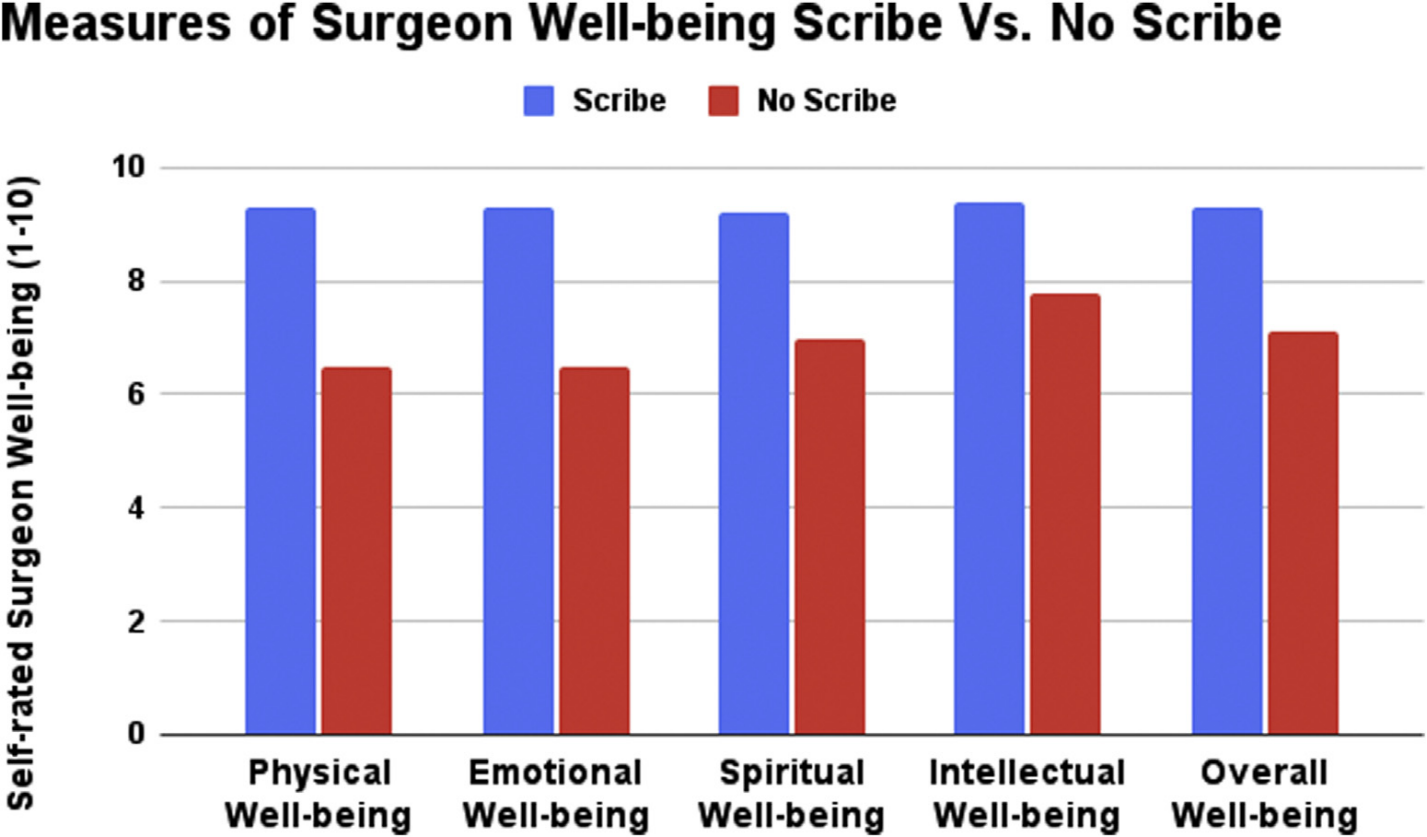
Surgeon self-rated well-being with scribes and without scribes.

There were 30 physician surveys filled out on scribe clinic days. The average time spent per half day of clinic spent documenting after the last patient was 25 minutes, on average, significantly less than the 104 minutes spent on nonscribe clinic days (*P* < .001). On a scale of 1 (strongly disagree) to 5 (strongly agree), surgeons reported a significantly greater score of 4.4 out of 5 (*P* < .001) for having “adequate time to perform patient education in clinic today,” and 4.1 out of 5 (*P* < .001) for “I had adequate time to teach medical students and trainees today.” The time spent documenting encounters throughout each half-day of clinic was estimated to be significantly less at 26 minutes on average (*P* < .001). The average SANE score was also significantly higher than the no-scribe clinic days at 89.3 (*P* < .001). The average scores on measures of well-being were all significantly higher when compared to the no-scribe clinic days. These well-being scores were physical well-being (9.3/10; *P* < .001), emotional well-being (9.3/10; *P* < .001), spiritual well-being (9.2/10; *P* < .001), intellectual wellbeing (9.4/10; *P* < .001), and overall well-being (9.3/10, *P* < .001) (Table 1, Figs 1 and 2).

**Table 1:** Orthopaedic Sport Surgeon Data on Clinic Days With Scribes and Without Scribes

Item	Overall (*n* = 62)	Scribe Present (*n* = 30)	No Scribe (*n* = 32)	*P* Value
I had adequate time to perform patient education in clinic today (1-Strongly Disagree to 5-Strongly Agree), mean (SD)	3.3 (1.4)	4.4 (0.5)	2.2 (1.1)	<.001
I had adequate time to teach medical students and trainees today (1-Strongly Disagree to 5-Strongly Agree), mean (SD)	3.0 (1.4)	4.1 (0.9)	2.0 (0.8)	<.001
Number of patients seen	19.8 (7.3)	22.0 (7.6)	17.8 (6.4)	.02
*Time*				
Time (minutes) spent documenting after last patient per half-day of clinic, mean (SD)	63.6 (77.6)	24.7 (30.9)	104.2 (90.1)	<.001
Total estimated time (minutes) spent documenting encounters throughout each half day of clinic, mean (SD)	57.2 (46.0)	25.5 (10.25)	86.8 (46.8)	<.001
*Measures of Well-Being*				
Physical well-being, mean (SD)	7.8 (2.3)	9.3 (0.9)	6.5 (2.4)	<.001
Emotional well-being, mean (SD)	7.8 (2.2)	9.3 (0.9)	6.5 (2.3)	<.001
Spiritual well-being, mean (SD)	8.1 (1.9)	9.2 (0.9)	7.0 (2.0)	<.001
Intellectual well-being, mean (SD)	8.6 (1.3)	9.4 (0.8)	7.8 (1.2)	<.001
Overall well-being, mean (SD)	8.1 (1.7)	9.3 (0.9)	7.1 (1.6)	<.001
Sane Score, mean (SD)	68.1 (24.5)	89.3 (7.0)	48.1 (16.9)	<.001

### Nurse Results

There were 42 nonscribe surveys completed by 3 nurses. The average number of patients seen per half-day of clinic was 24. Nurses answered yes on 30/42 surveys (71.4%) to “the surgeon was rushed today” and 32/42 (76.2%) answered yes to “the communication outside of the exam room was effective today.” The overall assessment of the clinic was 73.4 out of 100 on average.

There were 30 scribe surveys completed by 3 Nurses. The average number of patients seen per half-day of clinic was 27. Nurses answered yes on 8/30 surveys (26.7%) to “the surgeon was rushed today” and 29/30 (96.7%) answered yes to “the communication outside of the exam room was effective today.” The overall assessment of the clinic was significantly greater with a scribe present with a score of 87.7 out of 100 on average (*P* < .001) (Table 2). Additionally, the average time between the last patient leaving and the completion of nursing duties was significantly less on scribe clinic days (53.2 minutes no-scribe vs. 14.2 minutes scribe; *P* < .001).

**Table 2:** Nurses’ Ratings of Clinic with Scribes Versus Without Scribes

Item	Overall (*n* = 72)	Scribe Present (*n* = 30)	No Scribe (*n* = 42)	*P* Value
Overall assessment of clinic, mean (SD)	79.4 (14.0)	87.7 (10.1)	73.4 (13.4)	<.001
*Was the Surgeon Rushed or Running Late?*				
Yes (%)	38 (52.8%)	8 (26.7%)	30 (71.4%)	
No (%)	34 (47.2%)	22 (73.3%)	12 (28.6%)	
*Was Communication Outside of the Exam Room Effective?*				
TRUE (%)	61 (84.7%)	29 (96.7%)	32 (76.2%)	
FALSE (%)	11 (15.3%)	1 (3.3%)	10 (23.8%)	
*Time*				
Time (minutes) between last patient leaving and completing nursing duties, mean (SD)	36.6 (46.3)	14.2 (13.6)	53.2 (54.4)	<.001

### Patient Results

There were 631 nonscribe patients with an average age of 46.7, consisting of 284 female and 347 male patients. There was a nonresponse rate of 14%. Demographic information can be seen in Table 3.

**Table 3:** Patient Demographics

	Total Number of Patients (%)	Number of Patients With Scribes Present (%)	Number of Patients Without Scribes Present (%)
Overall	778	147	631
*Gender*			
Male	426 (54.8)	79	347
Female	352 (45.2)	68	284
Age			
<18	58 (7.5)	11	47
18-29	142 (18.2)	28	114
30-49	217 (27.9)	33	184
50-64	252 (32.4)	59	193
65+	109 (14.0)	16	93
Average (SD)	45.0	45.5	44.9
*Encounter Type*			
New patient	275 (39.0)	57	218
Follow up	412 (58.3)	76	336
Other	19 (2.7)	7	12

Patients rated their overall experience as 4.8 out of 5 on average. Patients typically arrived to their exam room 1.1 minutes before their appointment start time. Surgeons arrived to the room 19.2 minutes after rooming time and 18.1 minutes after official appointment time.

There were 147 scribe patients with an average age of 45.5, consisting of 68 female and 79 male patients. Patients rated their overall experience as 4.7 out of 5 on average. Patients typically arrived to their exam room 3.5 minutes before their appointment start time. Surgeons arrived to the room 18.6 minutes after rooming time and 15.1 minutes after official appointment time (Table 4). For the scribe and no-scribe groups, there was no significant difference between the average patient experience score (*P* = .27), time from patient appointment until exam room placement (*P* = .10), time from patient exam room placement until surgeon arrival (*P* = .67), or time from scheduled appointment time until surgeon arrival (*P* = .12).

**Table 4:** Patient Data Scribe vs. No Scribe

Item	Overall (*n* = 778)	Scribe Present (*n* = 147)	No Scribe (*n* = 631)	*P* Value
Patient experience score, mean (SD)	4.8 (0.5)	4.7 (0.5)	4.8 (0.5)	.27
*Wait Time*				
Time (minutes) from patient appointment time until exam room placement, mean (SD)	−1.5 (16.0)	−3.5 (14.0)	−1.1 (16.4)	.10
Time (minutes) from patient exam room Placement until surgeon arrival, mean (SD)	19.1 (14.7)	18.6 (14.9)	19.2 (14.6)	.67
Time (minutes) from scheduled appointment time until surgeon arrival, mean (SD)	17.6 (20.9)	15.1 (19.4)	18.1 (21.2)	.12

## Discussion

The use of scribes in a high-volume orthopaedic sports medicine clinic decreased the amount of physician documentation time, positively impacted physician well-being, improved nurses’ assessment of the clinic overall, with no resulting change in patient satisfaction. In total, orthopaedic sports surgeons spent a total of 191 minutes per half-day of clinic on average documenting throughout the day and using the computer with a scribe. When compared to a half-clinic day with a scribe, this amount of time decreases significantly from 191 minutes to 50 minutes, a 74% decrease. More specifically, the use of a scribe decreased the amount of documentation significantly from an average of 104 minutes to 25 minutes per half day of clinic, a 76% decrease in documentation time. The use of a scribe also had a positive impact on surgeon SANE scores and physician well-being. Surgeons reported having more time to educate students, residents, and patients when using a scribe. The use of a scribe improved nursing assessments of the clinic overall, provider communication, and sense of physician rushing. Patients also benefitted from scribes, with reduced rooming time and reduce waiting time for the surgeon. No significant difference was noted in patient clinic experience scores.

These findings are similar to a 2018 pilot study performed in an academic practice at the University of Chicago, where a single outsourced medical scribe was hired to assist in the clinics of six attending physicians in primary care on a rotating basis over the course of 3 months.^10^ The study found that the use of scribes decreased post-visit documentation time by half. These findings corroborate our findings, where documentation time spent by orthopaedic surgeons per half-day of clinic after the last patient decreased by 76% after implementation of scribes. Similarly, a study performed in primary care clinics showed that physicians with scribes reported less than 10 hours per week of charting versus 20-26 hours per week without scribes.^26^ The physicians in this study also reported that the time saved from using scribes was spent to engage more fully with patients and staff.^26^ Using scribes in high-volume orthopaedic sports clinics could help decrease post-visit documentation time, as noted by the substantial decrease in documentation time.

As there is increased time for surgeons to fully engage with patient and staff while using a scribe, it is not surprising that scribe use positively influences physician satisfaction and well-being. A recent study of emergency medicine physicians with scribes found physician satisfaction at their institution increased from 62nd percentile to 92nd percentile.^11^ Additionally, a separate study found that 100% of the 33 oncology physicians in their cohort strongly agreed that scribes improved their quality of life.^27^ A systematic review and meta-analysis of studies related to scribe use in the emergency department found that 14/16 studies reported increased provider satisfaction with scribes.^28^ Furthermore, a study of clinic days in urology found that 69% of physicians report statistically significant job satisfaction when working with a scribe, compared to 19% job satisfaction without scribes.^15^ Not surprisingly, in our study, we found that the average SANE score increased from 48 without scribes to 89 with scribes, along with a substantially increased self-rated physical, emotional, spiritual, intellectual, and overall well-being of the surgeons using scribes compared to not having scribes.

While we report improvement in physician well-being and decreased documentation time, the impact of scribes from a patient perspective also deserves exploration. Corroborating other studies examining patient satisfaction and scribe use in cardiology, otolaryngology, internal medicine, and urology, our study found that there was no significant impact of scribes on patients.^10^^,^^13^^,^^15^^,^^29^ This is an important finding, as some would argue that the use of scribes could decrease patient satisfaction or harm the overall patient experience. Some studies have even reported increased patient satisfaction among patients who are seen by physicians with scribes compared to physicians without scribes.^11^ This could be due to increased time spent with patients. A study of 129 physicians in an outpatient oncology practice found that 90% of physicians using scribes strongly agreed that they spent more time with patients and less time at the computer.^27^ In the 2018 Oxford study of scribes, there was a high degree of patient acceptance associated with the introduction of scribes and overall patient satisfaction remained high. We also found in our study that patient satisfaction remained high despite the use of scribes.

With patient satisfaction being reportedly neutral or positive with the use of scribes, the positive impact of scribes on patient satisfaction could be due to a variety of factors. Physicians in a 2018 pilot study for scribe use at an academic center reported that they felt less rushed, less distracted by the EHR, and more able to connect with patients. In exit interviews, one physician stated, “You had me at the first visit … first time in ten years I was able to truly focus on the patient without the distraction of EHR.” Others noted that they had “less sense of dread during busy clinics,” and it was “great to have my notes done so I could go home and have dinner with my family.” We also found in our study that others could notice the difference in surgeons, with nurses reporting that surgeons were much less rushed when using a scribe (71.4% of nurses reported rushed surgeons without the use of scribes to 26.7% with the use of scribes). Another randomized crossover study in primary care found that physicians reported significant improvement in patient interaction and clinical interactions during scribed-periods versus unscribed-periods.^30^

Our data show that physicians were able to see patients sooner when scribes were present. Surgeons arrived to the room 18.1 minutes after the official appointment time on clinical days without scribes compared to 15.1 with scribes. These results are further contextualized by a randomized controlled trial in foot and ankle orthopaedic surgery virtual scribes. The authors found that surgeons reported more time spent with the patient when working with a scribe at 14 minutes versus 11.4 minutes on average, and no significant difference in patient rating.^31^ A study in emergency medicine found a similar result, with door-to-doc time decreased from 74 minutes without a scribe to 62 minutes with scribe use.^11^

Trainees benefit from scribe use as well. This data showed that the use of scribes allowed for time to teach trainees, with an average rating on a scale of 1 (strongly disagree) to 5 (Strongly agree), surgeons without scribes reported a 2.0 out of 5 score for “I had adequate time to teach medical students and trainees today,” and 4.1 out of 5 score with the use of scribes. A separate study in dermatology about this subject found that 57% of attendings and 76% of trainees perceived that scribes increased the attendings’ direct teaching time, with 57% of physicians and 80% of trainees reported an improved overall education with the use of scribes compared to not using scribes.^32^ Also, scribes are often students applying for professional health programs, such as nursing, medical school, physician assistant school. These scribe experiences often provide valuable clinical experiences to help these students prepare for professional medical education programs.

Although our study does not report differences in productivity, it is important to note that scribes have also been reported to increase physicians’ productivity. A systematic review of scribe use in family medicine reported that both studies that measured physician productivity reported increase in work relative value units (wRVUs) with scribes.^19^ According to a study done at Hennepin County Medical Center, an academic hospital, scribes increased revenue for the emergency department and frequently increased revenue for subspecialty clinics.^33^ A study examining the productivity requirements of implementing a medical scribe program found that in orthopaedic surgery an average of 2.78 additional visits per day and 1.80 additional new patients per day are needed to cover the cost of scribes.^34^

In gastroenterology, a proof-of-concept study found that with the average time saved through use of a scribe, there was enough time at the end of the day to perform an additional procedure.^35^ Another study of pediatric emergency medicine found that patients per hour increased from 1.97 patients per hour to 2.21 patients per hour, and wRVUs increased from 2.55 wRVUs per hour to 4.27 wRVUs per hour.^36^ Another study of emergency medicine found that physician productivity increased from 2.3 to 3.2 patients per hour and the community emergency departments’ productivity increased from 241 to 336 wRVUs per hour pre- versus post-scribe implementation.^16^ A study of family medicine found that scribe implementation of two full-time scribes would cost $79,500 for both of them combined. The study found the projected increased revenue is more than $168,600 per year, more than enough to cover the use of scribes.^6^ In conjunction with increased revenue from scribe use, scribe use has even been found to lower staffing costs and an annual cost reduction compared to having no scribes in an orthopaedic trauma outpatient unit.^37^ The potential cost savings of scribe use in orthopaedic sports medicine clinics warrants further exploration, especially in the context of the rapidly changing financial and political landscape of orthopaedic surgery, with practice consolidation and decreasing reimbursement.^38^, ^39^, ^40^, ^41^

### Limitations

Our study is not without limitations. First, we had to exclude 268 patients because they did not fill out their survey completely or their answers were unclear (e.g., circling no answers or circling two answers for one question). Additionally, the number of surveys filled out with scribes is lower than the number of surveys without scribes due to the limited number of months we had access to scribes. Lastly, our surveys may not represent the broader physician, nurse, or patient experience.

### Conclusion

We found the use of scribes in a high-volume orthopaedic sports medicine clinic to have a favorable impact on physicians, nurses, and trainees. The use of a scribe also significantly reduced the time required by surgeons for documentation during clinic and at the end of each clinic day. Patients also reported no significant difference in patient clinic experience scores.

## Supplementary Data

ICMJE author disclosure forms
