# Supplementary material for: Medical Scribes in an Orthopedic Sports Medicine Clinic Improve Productivity and Physician Well-Being
Source: Arthrosc Sports Med Rehabil. 2022 Apr 8;4(3):e997–e1005. doi: 10.1016/j.asmr.2022.02.003 (PMC9210372; doi:10.1016/j.asmr.2022.02.003)
Supplement: Appendix [file mmc2.docx]

**Clinical Practice Innovation Program Data Collection**

Surgeon Assessment Form

**Surgeon: AC KE JT** **Date:**_____________ **Scribe Present**: Yes No

1. **I had adequate time to perform patient education in clinic today (circle one)**:

Strongly Disagree Disagree Neutral Agree Strongly Agree

1. **I had adequate time to teach medical students and trainees today** **(circle one):**

Strongly Disagree Disagree Neutral Agree Strongly Agree

1. **Today, my portion of daily clinic documentation was completed at**: __________________(time of day)
2. **Overall estimated time that I spent documenting encounters today**:__________________(hours/mins)
3. **‘SANE’ Score of my clinic today (subjective overall assessment )**: _____________________(0-100)


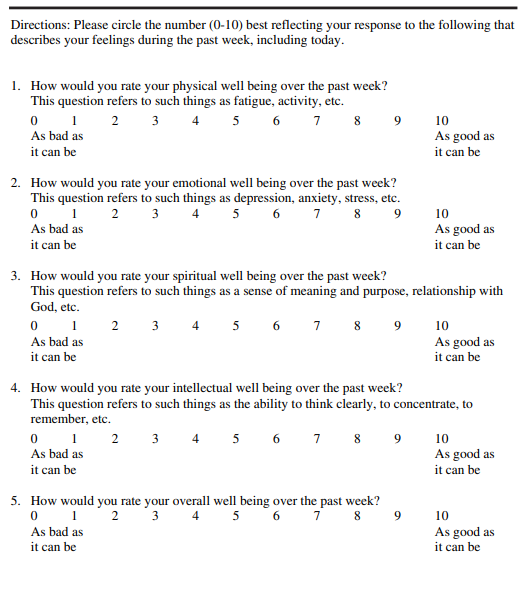


**Clinical Practice Innovation Program Data Collection**

Clinic RN Assessment Form

**Surgeon: AC KE JT** **Date:**_____________ **Scribe Present**: Yes No

1. **Number of actual patient encounters for this provider today: __________________________**
2. **Was the surgeon ‘rushed’ or ‘running late’ in clinic today?** Yes No Unsure
3. **Communication outside of the exam room today was effective.** True False Unsure
4. **All of the RN responsibilities related to this day of clinic were completed at:_____________(time)**
5. **My subjective overall assessment of clinic (0=Awful, 100=Awesome )**: __________________**(0-100)**

**Clinical Practice Innovation Program Data Collection**

Patient Assessment Form

**Date:**_____________ **Type of appointment**: New Patient Follow up visit Other

1. **Was a medical scribe present during your encounter with the surgeon today?**

**Yes No Unsure**

1. **If present, how did the scribe affect your clinic experience?**

**Improved No effect Impaired NA**

1. **Was the surgeon able to answer all your questions and explain your diagnosis?**

**Yes No NA**

1. **How would you rate your overall experience in clinic today?**

**Terrible Poor Okay Good Excellent**

1. **What time was your appointment today? __________________**
2. **When did you arrive in the exam room? __________________(approximate time)**
3. **When did the surgeon arrive in the exam room?_________________(approximate time)**
